# Cerebral and peripheral haemodynamics at rest and in response to orthostasis during 3 days of simulated heatwave

**DOI:** 10.1113/EP093589

**Published:** 2026-08-27

**Authors:** Alexander T. Friend, Samuel J. Oliver, Lydia L. Simpson, Carmen Possnig, Gabriella M. K. Rossetti, Jason T. Fisher, Lydia Tsoutsoubi, Konstantinos Mantzios, Urša Ciuha, Leonidas G. Ioannou, Igor B. Mekjavic, Justin S. Lawley

**Affiliations:** ^1^ Institute for Applied Human Physiology, School of Psychology and Sport Science, College of Medicine and Health Bangor University Bangor UK; ^2^ Bristol Medical School University of Bristol Bristol UK; ^3^ Department of Sports Science, Division of Performance Physiology and Prevention University of Innsbruck Innsbruck Austria; ^4^ Institute of Sport, Department of Sport and Exercise Sciences, Faculty of Science and Engineering Manchester Metropolitan University Manchester UK; ^5^ Department of Automatics, Biocybernetics and Robotics Jozef Stefan Institute Ljubljana Slovenia; ^6^ FAME Laboratory, School of Physical Education and Sport Science University of Thessaly Trikala Greece; ^7^ Institute of Mountain Emergency Medicine Eurac Research Bolzano Italy

**Keywords:** blood flow, cerebral, femoral, heat stress, heatwave, orthostasis, vascular

## Abstract

Acute heat stress reduces cerebral haemodynamics and orthostatic tolerance. However, the haemodynamic response to orthostasis during prolonged heat stress typical of heatwaves is unknown. We hypothesised that thermoregulatory‐induced elevations in peripheral haemodynamics during 3 days of simulated heatwave would exacerbate orthostatic‐induced reductions in cerebral haemodynamics observed in temperate conditions. Seven males (22 ± 1 years) maintained a standard work/rest ratio during temperate (∼25.4°C) and heatwave (∼35.4°C) conditions. Gastrointestinal core, mean skin and body (${\bar{T}}_{b}$) temperatures, haemoglobin concentration, mean arterial pressure (MAP), cerebral and peripheral haemodynamics (middle cerebral artery blood velocity (MCA_v_), internal carotid, vertebral, and superficial femoral artery blood flow (sFA_bf_)) were assessed at rest. MAP, middle cerebral artery vascular conductance index (MCA_VCi_), and superficial femoral artery vascular conductance (sFA_VC_) were measured during orthostasis caused by 60° head‐up tilt. Heatwave increased resting ${\bar{T}}_{b}$ (+0.76 ± 0.24°C, *P *< 0.001, *d = *3.02 vs. temperate), haemoglobin concentration (*P *= 0.004), sFA_bf_ (*P *= 0.001) and sFA (+34.0 ± 14.0 mL O_2_ min^−1^, *P *< 0.001, *d *= 2.61) and global cerebral (+21.3 ± 16.8 mL O_2_ min^−1^, *P *= 0.009, *d = *0.71) oxygen delivery, whereas MAP (*P *= 0.729) and MCA_v_ (*P *= 0.687) were unchanged. During tilt, MCA_VCi_ reductions were comparable between conditions (day × posture interaction, *P *= 0.959; posture main effect, −0.19 ± 0.06 cm s^−1^ mmHg^−1^, *P *< 0.001, *d *= 2.05) whilst the heatwave‐induced elevation in sFA_VC_ was reduced (interaction, *P *= 0.008). In conclusion, orthostatic‐induced reductions in cerebral haemodynamics were not exacerbated across heatwave days, whereas the heatwave‐induced elevation in resting supine peripheral haemodynamics was attenuated during orthostasis. Additionally, resting global cerebral oxygen delivery was increased during the latter heatwave days. These divergent cerebral and peripheral haemodynamic responses in males suggest cerebral perfusion and oxygen delivery are prioritised during prolonged heat stress.

## Abstract (highlights)

- **What is the central question of this study?**
  How does prolonged heat stress, typical of a heatwave, affect cerebral and peripheral vascular haemodynamics at rest and in response to orthostasis?
- **What is the main finding and its importance?**
  A 3‐day simulated heatwave increased resting supine leg blood flow and oxygen delivery, and cerebral oxygen delivery in males. The orthostatic‐induced reduction in cerebral haemodynamics was not exacerbated by the heatwave, whereas the heatwave‐induced elevation in supine leg haemodynamics was attenuated. These divergent cerebral and peripheral haemodynamic responses in males suggest cerebral perfusion and oxygen delivery are prioritised during prolonged heat stress.

## 1 INTRODUCTION

Acute (<12 h) heat stress decreases cerebral perfusion (Fan et al., 2008; Gibbons et al., 2021; Nelson et al., 2011; Ogoh et al., 2013; Ross et al., 2012), exacerbates orthostatic‐induced reductions in cerebral perfusion (Lucas et al., 2013; Wilson et al., 2006), reduces tolerance to orthostatic challenges (Lind et al., 1968; Minson et al., 1999), and is associated with increased syncope and occupational accidents (Parsons et al., 2023; Ramsey et al., 1983; Tawatsupa et al., 2013; Varghese et al., 2018). The most likely mechanisms to explain the compromised cerebral perfusion during acute heat stress and orthostasis is inadequate cerebral perfusion pressure and a fall in cerebral blood flow through the combination of heat‐ and orthostatic‐induced central hypovolaemia, cerebral vasoconstriction induced by hyperventilation and reductions in end‐tidal CO_2_, and increases in sympathetic excitation (Crandall & Wilson, 2015; Schlader et al., 2016). Assessing peripheral and cerebral haemodynamics is key to understanding the balance between thermoregulatory demands and cerebrovascular stability (Nelson et al., 2011; Wilson et al., 2002). To date, cerebral and peripheral haemodynamic changes to heat stress have mostly been examined during rapid acute (Fan et al., 2008; Nelson et al., 2011; Ogoh et al., 2013; Ross et al., 2012; Wilson et al., 2006) or intermittent heat stress (Fujii et al., 2015). In contrast, the effects of prolonged (>24 h) heat stress, such as that experienced during heatwaves, on cerebral and peripheral haemodynamics are largely unexplored.

Whilst prolonged heat stress may initially trigger similar physiological responses to acute heat stress, the slower onset and prolonged elevation in ambient temperature may allow time for heat acclimation and attenuated cerebral and peripheral haemodynamic responses. Indeed, 3 days of continuous heat exposure caused rapid adaptive cardiovascular responses that reduced physiological strain (i.e., attenuation of heat‐induced elevations in core temperature, skin temperature and heart rate) and maintained work‐related cognitive performance (Ioannou et al., 2021). Determining the effects of more prolonged heat stress on cerebral and peripheral haemodynamic responses at supine rest and in response to orthostasis is important to help develop targeted countermeasures to reduce heatwave‐related morbidity and mortality (Basu & Samet, 2002; Guo et al., 2018; Lin et al., 2013; Moghadamnia et al., 2017; Tsoutsoubi et al., 2021).

This study aimed to examine the cerebral and peripheral haemodynamic responses at supine rest and to a 60° head‐up tilt orthostatic challenge each day of a 3‐day simulated heatwave. We hypothesised that on day 1 of the simulated heatwave, prolonged heat stress would increase peripheral blood flow but reduce resting cerebral blood flow and exacerbate orthostatic‐induced reductions in cerebral haemodynamics. We also hypothesised that as the simulated heatwave progressed, due to heat acclimation and associated thermoregulatory and cardiovascular adaptations (Ioannou et al., 2021), peripheral blood flow would remain elevated during the heatwave, whereas the reduction in cerebral haemodynamics observed on day 1 would be attenuated.

## 2 METHODS

### 2.1 Study design

The current study was conducted at the Planetary Habitat facility (a European Space Agency ground‐based research facility) at the Olympic Sport Centre Planica (Rateče, Planica) as part of the European Commission Horizon 2020 project ‘Heat Shield’. This project investigated the effect of heatwaves on the health, well‐being and labour productivity of male workers. Further experimental design details, including sample size, and findings on the impact of a simulated heatwave on daytime physiology and productivity have been reported elsewhere (Fisher et al., 2022; Ioannou et al., 2021, 2024). This sample size was also sufficient to detect a similar magnitude of difference in cerebral blood velocity after orthostasis between heat stress and normothermia as previously observed (Wilson et al., 2006), and when using standard α (0.05) and power (0.8), and at least a 0.6 correlation among repeated measures, which is at the lower end of previous reports (Friend et al., 2025; Shariffi et al., 2023).

### 2.2 Ethical approval

Ethical approval for this study was obtained from the National Committee for Medical Ethics at the Ministry of Health of the Republic of Slovenia (approval no. 0120‐402/2020/4: 20 October 2020) and was conducted following the standards of the *Declaration of Helsinki* 2013, except for registration in a database, with written informed consent obtained from all participants.

### 2.3 Participants

Seven healthy White male participants were recruited for this study (age: 21.5 ± 1.2 years; height: 180 ± 5.6 cm; body mass: 81.5 ± 14.5 kg; fat mass: 22.5 ± 7.9%). Participants engaged in regular physical activity, were non‐smokers, and were free from cardiovascular, haematological and neurological disease.

### 2.4 Experimental procedures

Participants were confined to designated areas of the centre for 9 days, during which ambient temperature and relative humidity were continuously monitored and regulated to simulate three 3‐day periods: thermoneutral pre‐heatwave (days 1–3), heatwave (days 4–6) and post‐heatwave (days 7–9) (Ioannou et al., 2021). The onset of the heatwave occurred between midnight (24.00 h) on day 3 until midnight (24.00 h) on day 6. The daily 24 h ambient temperature was adjusted for working (09.00−18.00 h) and resting (18.00−09.00 h) hours for simulated temperate (work ∼25.4°C and rest ∼22.3°C) and heatwave (work ∼35.4°C and rest ∼26.3°C) conditions to reflect normal diurnal changes in ambient temperature (Ioannou et al., 2021). To assess a separate project aim related to work productivity, all participants followed a standardised work/rest schedule consisting of two 40‐min sessions of low‐intensity exercise and two 1‐h period of simulated work performed in overalls at 10.00 h and 16.00 h interspersed with seated passive heat exposure, identical meal plans (lunch 12.00 h, dinner 18.20 h), and water consumed ad libitum (Ioannou et al., 2021). The orthostatic stress challenges consisted of a passive 60° head‐up tilt for 10 min and were conducted between 13.00 and 16.00 h each day (i.e., during work temperature) with each participant tested at the same time of day during both temperate and heatwave conditions.

### 2.5 Experimental measurements

#### 2.5.1 Thermoregulatory measurements

Core temperature was measured in the gastrointestinal tract (*T*_gi_) by ingestible telemetric pills (Bodycap, Caen, France), with a new pill ingested at the same time (07.00 h) every morning. Skin temperature at the chest, bicep, thigh and calf was measured from thermistors (iButtons type DS1921H, Maxim/Analog Devices, Inc., Wilmington, MA, USA) and used to calculate a mean skin temperature (${\bar{T}}_{\mathrm{sk}}$) using the weighted average (Ramanathan, 1964): ${\bar{T}}_{\mathrm{sk}}$ = 0.3(*T*_chest_ + *T*_bicep_) + 0.2(*T*_thigh_ + *T*_calf_). Minute‐by‐minute measurements of *T*_gi_ (09.00–07.00 h) and *T*_sk_ (23.00–21.00 h) were collected for 22 h each day and time‐aligned. As an indicator of whole‐body thermal status, mean body temperature (${\bar{T}}_{b}$) was estimated using the proposed weighted average formula (Burton, 1935; Lenhardt & Sessler, 2006): ${\bar{T}}_{b}$ = (0.64 × *T*_gi_) + (0.36 × ${\bar{T}}_{\mathrm{sk}}$).

#### 2.5.2 Cardiorespiratory measurements

Beat‐to‐beat heart rate and blood pressure were measured by Lead II of an electrocardiogram and finger arterial clamping (volume‐clamp method) with infrared photoplethysmography, respectively (Finapres Nova; Finapres Medical Systems BV, Amsterdam, The Netherlands). Measurements of systolic blood pressure, diastolic blood pressure and mean arterial pressure (MAP) were calculated from the finger arterial waveform that was calibrated to the average of two automated brachial blood pressure measurements on each day (Tango+, SunTech, Morrisville, NC, USA). The Model Flow algorithm recorded approximations of stroke volume, cardiac output and total peripheral resistance (TPR = MAP/Cardiac output). The partial pressure of end‐tidal carbon dioxide ($P_{\mathrm{ETC}O_{2}}$) was continuously recorded from the mouth using a nose clip by a gas analyser (ML206, ADInstruments, Dunedin, New Zealand).

#### 2.5.3 Transcranial doppler ultrasound

Blood velocity of the middle cerebral artery (MCA_v_) was measured by transcranial Doppler ultrasound using a 2 MHz probe placed over the left transtemporal windows and secured in place via an adjustable headpiece (PMD150, Spencer Technologies, Seattle, WA, USA). Insonation of the MCA was achieved using standardised procedures with probe position, signal depth and gain settings recorded and replicated between sessions (Willie et al., 2011).

Cardiorespiratory and transcranial Doppler ultrasound variables were all acquired continuously during rest and head‐up tilt at 400 Hz using an analog‐to‐digital converter (PowerLab 16/30; ADInstruments) and interfaced on a computer in real time using LabChart software (LabChart Pro V8.3.1, ADInstruments).

#### 2.5.4 Duplex ultrasound

Blood flow of the superficial femoral artery (sFA_bf_), internal carotid artery (ICA_bf_) and vertebral artery (VA_bf_) was collected on the right side of the body using duplex ultrasound with a 15 MHz linear transducer (uSmart 3300, Terason, Burlington, MA, USA). High‐resolution images of vessel diameter were acquired using B‐mode imaging whilst pulse wave mode was used to simultaneously measure the Doppler velocity spectra. Care was taken to ensure the positioning of the Doppler gate encompassed the entire vessel with a 60° angle of insonation as per recommendations (Thomas et al., 2015). sFA_bf_ was measured at least 2 cm distal to the femoral bifurcation to avoid turbulent flow. ICA_bf_ was measured at least 1.0−1.5 cm distal to the carotid bifurcation and VA_bf_ was measured between C3 and the subclavian artery. All duplex ultrasound measurements were collected by a single operator (J.S.L.). Subsequently, sFA_bf,_ ICA_bf_ and VA_bf_ at supine rest were measured in sequence, whereas during head‐up tilt only sFA_bf_ was collected. Acquired duplex ultrasound images were captured using custom edge‐detection tracking software and stored for subsequent offline analysis by an investigator blinded to the condition of the experimental trials.

### 2.6 Data analysis

Data are presented as an average from day 2 and day 3 of temperate conditions and from day 4 (HW1), day 5 (HW2) and day 6 (HW3) of the simulated heatwave condition. Blood flow was calculated from concurrent duplex ultrasound measurements of vessel diameter and time‐averaged mean velocity (TAMv) as: blood flow (mL min^−1^) = TAMv (cm s^−1^) × [π × (mean artery diameter (cm)/2)^2^] × 60. Heart rate, stroke volume, cardiac output, MAP, TPR, $P_{\mathrm{ETC}O_{2}}$, MCA_v_ and sFA_bf_ were calculated from the final 2 min of the 10‐min supine rest period and during each subsequent 2‐min period of the 10‐min head‐up tilt. ICA_bf_ and VA_bf_ were measured during the final 2 min of supine rest only. Global cerebral blood flow (gCBF) was calculated as (ICA_bf_ × 2)+(VA_bf_ × 2). Oxygen delivery ($D_{O_{2}}$ [mL min^−1^] = blood flow × content of arterial oxygen [$C_{aO_{2}}$]) was estimated for MCAv (index), sFA_bf,_ ICA_bf,_ VA_bf_, and gCBF where $C_{aO_{2}}$ (mL dl^−1^) = (Haemoglobin (Hb) × 1.39) × (arterial oxygen saturation [$S_{aO_{2}}$]) + (0.003 × partial pressure of arterial oxygen [$P_{aO_{2}}$]); Hb was measured by a ABL80 FLEX CO‐OX blood gas analyser (Radiometer Medical, Brønshøj, Denmark) in finger capillary blood collected on temperate day 3 and HW3, and $S_{aO_{2}}$ and $P_{aO_{2}}$ were estimated as 99% and 100 mmHg, respectively. Vascular conductance (VC = blood flow/MAP) or index (VCi = blood velocity/MAP) were calculated for sFA_bf,_ ICA_bf,_ VA_bf_ and MCA_v_ as appropriate.

### 2.7 Statistical analysis

Statistical analysis was conducted using SPSS Statistics v27 (IBM Corp., Armonk, NY, USA). All cardiorespiratory and haemodynamic variables were analysed by linear mixed model (LMM). For variables at supine rest, the fixed effect of interest was day with participant included as a random effect. For variables during head‐up tilt, the fixed effects were day and posture (rest vs. 10‐min post‐tilt), adding participant as a random effect, and the primary outcome of interest was the interaction of day × posture. Fisher's least significant difference pairwise comparisons were conducted where significant interactions or main effects were detected. Values are means ± standard deviation and statistical significance was set at *P* < 0.05. Effect sizes for *post hoc* comparisons were also reported to aid interpretation using the conventional criterion for small (*d* = 0.2), medium (*d* = 0.5) and large (*d* = 0.8) effects (Cohen, 1988), and mean differences are provided with 95% confidence intervals (CI). Repeated‐measures correlation analysis was used to determine relationships between ${\bar{T}}_{b}$ and cardiovascular or cerebrovascular variables using a publicly accessible application (Bakdash & Marusich, 2017; Marusich & Bakdash, 2021).

## 3 RESULTS

### 3.1 Thermoregulatory responses

The thermoregulatory responses at rest, and in response to stepping exercise and simulated assembly line tasks, to 3 days of simulated heatwave have been extensively described (Ioannou et al., 2021, 2024). In brief, on day 1 of the heatwave, increases in ${\bar{T}}_{b}$ (Temperate vs. HW1 +0.76 ± 0.24°C, 95% CI: +0.56 to +0.97°C, *P *< 0.001, *d = *3.02, Table 1), ${\bar{T}}_{\mathrm{sk}}$ (Temperate vs. HW1 +1.79 ± 0.51°C, 95% CI: +1.43 to +2.16°C, *P* < 0.001, *d *= 3.01) and *T*_gi_ (Temperate vs. HW1 +0.18 ± 0.19°C, 95% CI: −0.06 to +0.38°C, *P* = 0.056, *d = *1.02) indicated acute heat stress in participants, while by day 3, adaptation was evident by a return in *T*_gi_ to temperate values despite further increases in ${\bar{T}}_{\mathrm{sk}}$.

**TABLE 1:** Thermoregulatory and cardiorespiratory responses in males at supine rest during temperate and 3 days of simulated heatwave conditions.

	Temperate	HW1	HW2	HW3	*P*
${\bar{T}}_{b}$ (°C)	36.0 ± 0.2	36.7 ± 0.3^*^	36.8 ± 0.2^*^	36.8 ± 0.2^*^	**<0.001**
*T*_gi_ (°C)	37.3 ± 0.2	37.5 ± 0.2	37.4 ± 0.1	37.3 ± 0.3	0.095
${\bar{T}}_{\mathrm{sk}}$ (°C)	33.5 ± 0.6	35.3 ± 0.6^*^	35.7 ± 0.3^*^	35.8 ± 0.4^*‡^	**<0.001**
Heart rate (bpm)	67 ± 11	76 ± 12^*^	76 ± 13^*^	68 ± 12^‡#^	**<0.001**
Stroke volume (mL)	72.4 ± 17.6	76.8 ± 27.6	70.2 ± 16.5	87.7 ± 17.2	0.066
Cardiac output (L min^−1^)	4.74 ± 0.65	5.70 ± 1.77	5.28 ± 1.60	5.90 ± 1.25	0.123
MAP (mmHg)	89 ± 8	89 ± 8	93 ± 9	90 ± 14	0.729
TPR (mmHg mL min^−1^)	20.1 ± 4.6	17.2 ± 6.4	19.0 ± 6.1	15.8 ± 3.9	0.238
$P_{\mathrm{ETC}O_{2}}$ (mmHg)	37.9 ± 1.0	37.7 ± 1.3	38.3 ± 1.3^‡^	38.9 ± 1.4^*‡^	**0.002**
MCA_v_ (cm s^−1^)	65.1 ± 6.9	60.8 ± 9.1	62.6 ± 10.8	61.8 ± 6.0	0.687
ICA_bf_ (mL min^−1^)	363.3 ± 60.9	415.5 ± 103.6	388.5 ± 49.7	401.0 ± 79.6	0.197
VA_bf_ (mL min^−1^)	96.2 ± 28.5	121.4 ± 48.7	113.3 ± 36.2	116.1 ± 41.7	0.497
$\mathrm{ICA}D_{O_{2}}$ (mL O_2_ min^−1^)	75.8 ± 13.7	87.8 ± 19.8	82.5 ± 11.3	85.2 ± 17.1	0.106
$\mathrm{VA}D_{O_{2}}$ (mL O_2_ min^−1^)	19.9 ± 5.5	25.5 ± 9.5	23.9 ± 7.2	24.4 ± 8.1	0.362

Values are means ± standard deviation. *Post hoc* comparisons: ^*^ *P* < 0.05 vs. temperate; ^‡^ *P* < 0.05 vs. HW1; ^#^ *P* < 0.05 vs. HW2.

Abbreviations: HW1, heatwave day 1; HW2, heatwave day 2; HW3, heatwave day 3; ICA_bf_, internal carotid artery blood flow; $\mathrm{ICA}D_{O_{2}}$, internal carotid artery oxygen delivery; MAP, mean arterial pressure; MCA_v_, middle cerebral artery blood velocity; $P_{\mathrm{ETC}O_{2}}$, partial pressure of end‐tidal carbon dioxide; ${\bar{T}}_{b}$, mean body temperature; *T*_gi_, gastrointestinal core temperature; ${\bar{T}}_{\mathrm{sk}}$, mean skin temperature; TPR, total peripheral resistance; VA_bf_, vertebral artery blood flow; $\mathrm{VA}D_{O_{2}}$, vertebral artery oxygen delivery.

### 3.2 Cardiorespiratory and haemodynamic responses

**FIGURE 1:**
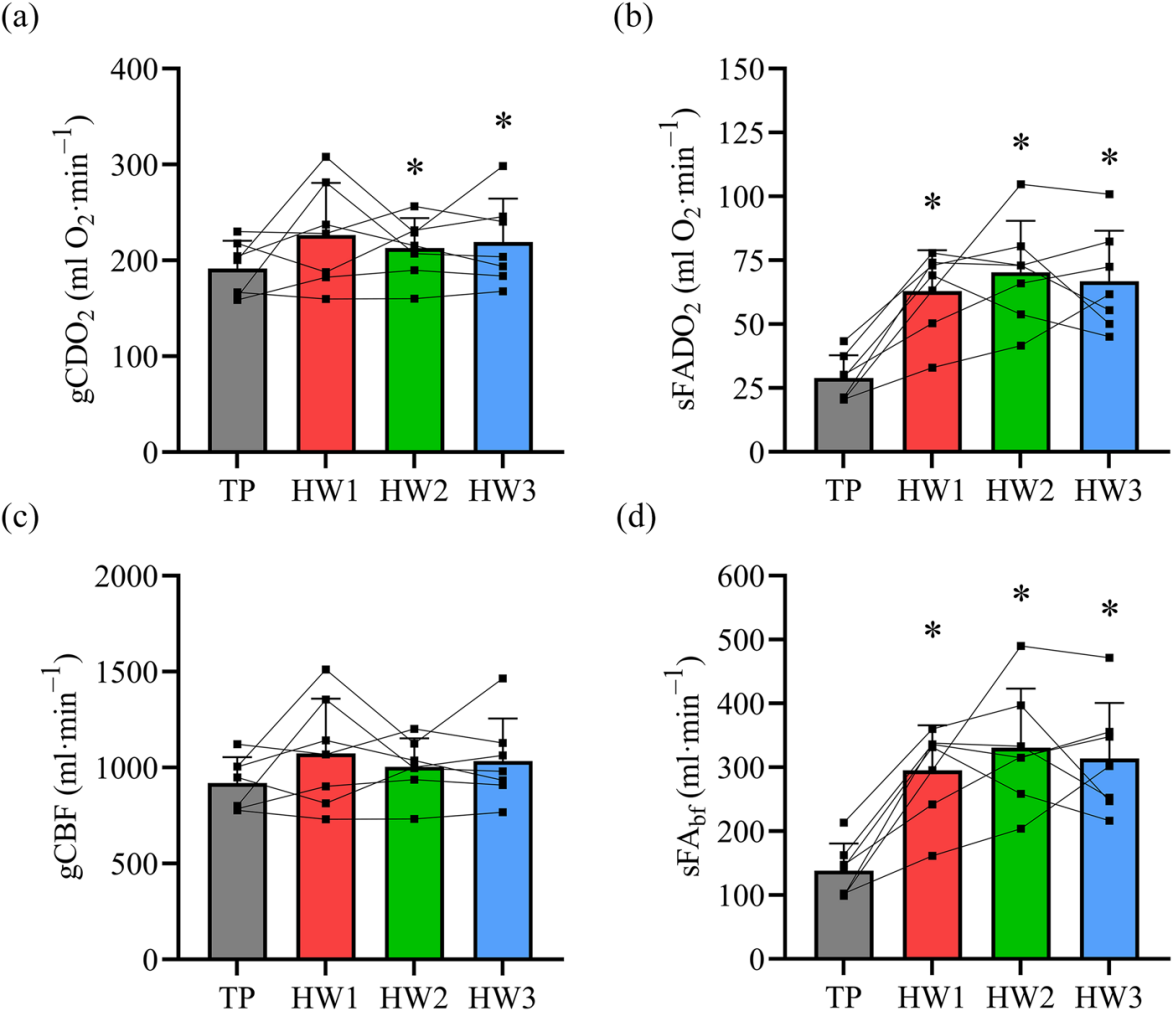
Cerebral and peripheral haemodynamic responses in males at supine rest during temperate and 3 days of simulated heatwave conditions. Global cerebral (internal carotid + vertebral artery) oxygen delivery (a; $\mathrm{gC}D_{O_{2}}$, mL O_2_ min^−1^, *P* = 0.027), superficial femoral artery oxygen delivery (b; $\mathrm{sFA}D_{O_{2}}$, mL O_2_ min^−1^, *P* < 0.001), global cerebral blood flow (c; gCBF, mL min^−1^, *P* = 0.061) and superficial femoral artery blood flow (d; sFA_bf_, mL min^−1^, *P* < 0.001) during temperate conditions (grey) and 3 days of simulated heatwave (heatwave day 1 [HW1], red bar; heatwave day 2 [HW2], green; heatwave day 3 [HW3], blue). Linear mixed model analysis was used to examine the effect of day for each variable. *P < 0.05 vs. temperate. Values are means ± standard deviation with individual data points.

Increases in heart rate, sFA_bf_ and $\mathrm{sFA}D_{O_{2}}$ during supine rest on day 1 of the simulated heatwave, compared with temperate condition, paralleled the thermoregulatory responses to indicate acute heat stress in participants (Temperate vs. HW1: heart rate +9 ± 10 bpm, 95% CI: +1 to +16 bpm, *P* = 0.038, *d = *0.75; sFA_bf_ +156.7 ± 62.3 mL min^−1^, 95% CI: +97.1 to +215.7 mL min^−1^, *P* = 0.001, *d = *2.70; $\mathrm{sFA}D_{O_{2}}$ +34.0 ± 14.0 mL O_2_ min^−1^, 95% CI: +21.2 to +46.8 mL O_2_ min^−1^, *P* < 0.001, *d = *2.61; Table 1 and Figure 1). A return of heart rate to that observed in temperate conditions by day 3 also evidenced adaptation to the simulated heatwave. However, sFA_bf_ and $\mathrm{sFA}D_{O_{2}}$ remained elevated on day 2 and day 3 of the simulated heatwave compared to the temperate condition (sFA_bf_ Temperate vs. HW2 +192.3 ± 98.0 mL min^−1^, 95% CI: +118.3 to +266.3 mL min^−1^, *P* < 0.001, *d* = 2.67; Temperate vs. HW3 +175.1 ± 109.2 mL min^−1^, 95% CI: +97.1 to +253.1 mL min^−1^, *P* = 0.001, *d* = 2.67; and $\mathrm{sFA}D_{O_{2}}$ Temperate vs. HW2 +41.5 ± 21.3 mL O_2_ min^−1^, 95% CI: +25.6 to +57.2 mL O_2_ min^−1^, *P* < 0.001, *d* = 2.67; Temperate vs. HW3 +37.9 ± 23.6 mL O_2_ min^−1^, 95% CI: +20.8 to +55.0 mL O_2_ min^−1^, *P* = 0.001, *d* = 2.47).

Hb concentration was increased by day 3 of heatwave compared to temperate conditions (Temperate vs. HW3 14.9 ± 1.4 g dl^−1^ vs. 15.2 ± 0.7 g dl^−1^, +0.29 ± 0.20 g dl^−1^, 95% CI: +0.12 to +0.45 g dl^−1^, *P* = 0.004, *d *= 0.38). $P_{\mathrm{ETC}O_{2}}$ was unchanged on day 1 of the heatwave, compared to the temperate condition, but was increased on day 2 (HW2 vs. HW1 +0.65 ± 0.86 mmHg, 95% CI: +0.03 to +1.28 mmHg, *P* = 0.049, *d *= 0.49) and day 3 of the simulated heatwave (HW3 vs. HW1 +1.26 ± 0.56 mmHg, 95% CI: +0.77 to +1.80 mmHg, *P* = 0.003, *d *= 0.92). Whilst the main effects of condition were statistically non‐significant (*P *= 0.066 and *P* = 0.123), stroke volume and cardiac output were increased by day 3 of the simulated heatwave with large effect sizes (Temperate vs. HW3: stroke volume +15.3 ± 13.7 mL, 95% CI: +2.44 to +28.2 mL, *P *= 0.027, *d *= 0.88; cardiac output +1.16 ± 1.52 L min^−1^, 95% CI: +0.69 to +2.25 L min^−1^, *P *= 0.039, *d *= 1.17).

Resting gCBF was similar during the simulated heatwave and temperate conditions (main effect of condition *P* = 0.061, Figure 1; Temperate vs. HW1 +154.7 ± 275.0 mL min^−1^,  95% CI: −37.4 to +346.9 mL min^−1^, *P* = 0.101, *d* = 0.69; Temperate vs. HW2 +84.5 ± 80.4 mL min^−1^, 95% CI: +17.9 to +151.2 mL min^−1^, *P* = 0.020, *d* = 0.59; Temperate vs. HW3 +115.1 ± 176.0 mL min^−1^, 95% CI: −16.3 to 246.5 mL min^−1^, *P* = 0.079, *d* = 0.63); however, due to an increase in Hb, $\mathrm{gC}D_{O_{2}}$ increased on day 2 and 3 of heatwave compared to temperate ($\mathrm{gC}D_{O_{2}}$ Temperate vs. HW1 +35.0 ± 57.7 mL O_2_ min^−1^, 95% CI: −5.4 to +75.4 mL O_2_ min^−1^, *P* = 0.081, *d* = 0.80; Temperate vs. HW2 +21.3 ± 16.8 mL O_2_ min^−1^, 95% CI: +7.3 to +35.4 mL O_2_ min^−1^, *P* = 0.009, *d* = 0.71; Temperate vs. HW3 +27.7 ± 35.7 mL O_2_ min^−1^, 95% CI: +1.0 to +54.4 mL O_2_ min^−1^, *P* = 0.044, *d* = 0.73). MAP (main effect of condition, *P* = 0.729), TPR (*P* = 0.238), MCA_v_ (*P* = 0.687), diameter of the ICA (*P* = 0.494) and VA (*P* = 0.170), blood velocity of the ICA (*P* = 0.200) and VA (*P* = 0.826), ICA_bf_ (*P* = 0.197), VA_bf_ (*P* = 0.497), $\mathrm{ICA}D_{O_{2}}$ (*P* = 0.111), and $\mathrm{VA}D_{O_{2}}$ (*P* = 0.384) were similar during the simulated heatwave and temperate condition.

For all days (i.e., temperate, HW1, HW2, HW3), relationships between the absolute value of ${\bar{T}}_{b}$ with the absolute value of heart rate (*r*_rm_ = 0.44, *P *= 0.04), $\mathrm{sFA}D_{O_{2}}$ (*r*_rm_ = 0.79, *P* < 0.001), sFA_bf_ (*r*_rm_ = 0.78, *P* < 0.001), $\mathrm{gC}D_{O_{2}}$ (*r*_rm_ = 0.43, *P* < 0.05) were found, whereas relationships with gCBF (*r*_rm_ = 0.38, *P* = 0.08), ICA_bf_ (*r*_rm_ = 0.36, *P* = 0.10), VA_bf_ (*r*_rm_ = 0.35 *P* = 0.11), MCA_v_ (*r*_rm_ = −0.37, *P* = 0.09) and $P_{\mathrm{ETC}O_{2}}$ (*r*_rm_ = 0.28, *P* = 0.20) did not reach statistical significance. For only the heatwave days (i.e., HW1, HW2, HW3), no significant relationships were found between ${\bar{T}}_{b}$ and cardiorespiratory or cerebrovascular variables (i.e., heart rate, MAP, $P_{\mathrm{ETC}O_{2}}$, gCBF, ICA_bf_, VA_bf_, MCA_v_, sFA_bf_, all *P* > 0.05).

### 3.3 Cardiorespiratory and haemodynamic responses to head‐up tilt

Cardiorespiratory responses to head‐up tilt did not differ between days (all day × posture interactions, *P *> 0.05), indicating that the magnitude of orthostatic stress was similar between temperate and simulated heatwave conditions. Indeed, irrespective of day, head‐up tilt elicited reductions in stroke volume (main effect of posture, −28.1 ± 10.1 mL, 95% CI: −36.3 to −18.8 mL, *P* < 0.001, *d* = 2.36), cardiac output (−0.65 ± 0.39 L min^−1^, 95% CI: −1.33 to −0.63 mL min^−1^, *P* = 0.073, *d* = 0.88) and $P_{\mathrm{ETC}O_{2}}$ (−3.06 ± 1.18 mmHg, 95% CI: −3.55 to −2.59 mmHg, *P* < 0.001, *d* = 3.11), with concomitant increases in heart rate (+27 ± 9 bpm, 95% CI: +20 to +33 bpm, *P* < 0.001, *d* = 2.29), MAP (+13 ± 4 mmHg, 95% CI: +9 to +17 mmHg, *P* < 0.001, *d* = 1.82) and TPR (+6.08 ± 3.66 mmHg l min^−1^, 95% CI: +2.79 to +9.12 mL min^−1^, *P* = 0.001, *d* = 1.41).

**FIGURE 2:**
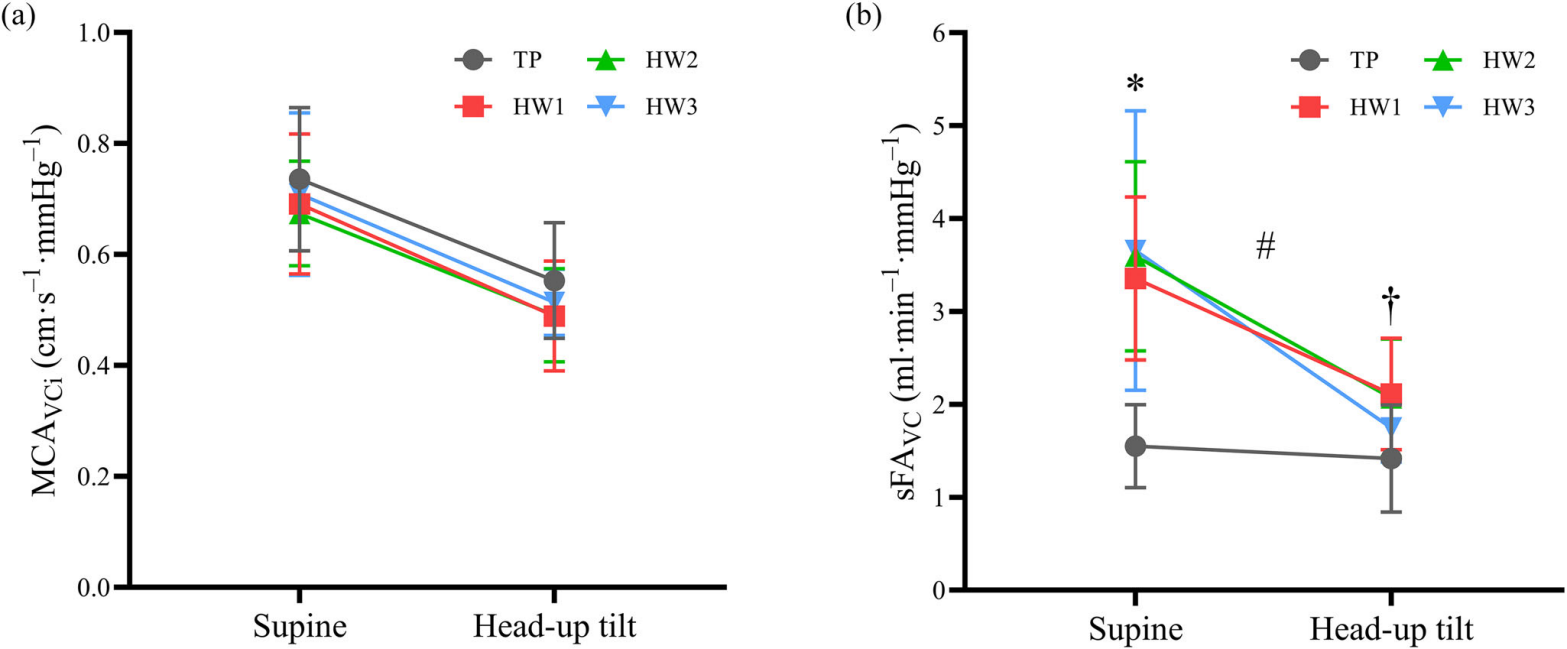
Cerebral and peripheral haemodynamic responses in males to 60° head‐up tilt during temperate and 3 days of simulated heatwave conditions. Middle cerebral artery vascular conductance index (a; MCA_VCi_, cm s^−1^, interaction: *P* = 0.959) and superficial femoral artery vascular conductance (b; sFA_VC_, mL min^−1^, *P* = 0.008) in temperate (grey) and after 3 days of heatwave conditions (heatwave day 1 [HW1], red; heatwave day 2 [HW2], green; heatwave day 3 [HW3], blue). Linear mixed model analysis was used to examine the day × posture (supine rest or 10 min post‐tilt) interaction for each variable. ^*^ *P* < 0.05 temperate vs. all HW days at supine. ^#^ *P* < 0.05 supine vs. head‐up tilt for HW days. ^†^ *P* < 0.05 temperate vs. HW1 and HW2 at head‐up tilt. Values are means ± standard deviation.

Head‐up tilt‐induced reductions in MCA_VCi_ (main effect of posture, −0.19 ± 0.06 cm s^−1^ mmHg^−1^, 95% CI: −0.22 to −0.16 cm s^−1^ mmHg^−1^, *P* < 0.001, *d* = 2.05) were not different between temperate and simulated heatwave conditions (day × posture interaction, *P* = 0.959; Figure 2). In contrast, the sFA_VC_ response to head‐up tilt was dependent on the conditions (day × posture interaction, *P* = 0.008, Figure 2b). In the temperate condition, sFA_VC_ did not change 10 min after head‐up tilt compared to rest (−0.11 ± 0.43 mL min^−1^ mmHg^−1^, 95% CI: −0.44 to +0.66 mL min^−1^ mmHg^−1^, *P* = 0.664, *d *= 0.21). In contrast, sFA_VC_ was reduced significantly 10 min after head‐up tilt during heatwave days (Tilt vs. supine: HW1 −1.24 ± 1.36 mL min^−1^ mmHg^−1^; HW2 −1.53 ± 0.76 mL min^−1^ mmHg^−1^; HW3 −1.91 ± 1.19 mL min^−1^ mmHg^−1^, 95% CI range: −3.09 to −0.44 mL min^−1^ mmHg^−1^, all *P* < 0.01, *d *= 1.65 to 1.80), indicating peripheral vasoconstriction. During head‐up tilt on days 1 and 2 of the heatwave, absolute sFA_VC_ was higher (Temperate vs. HW1 2.11 ± 0.60 mL min^−1^ mmHg^−1^, 95% CI: +0.08 to +1.27 mL min^−1^ mmHg^−1^, *P* = 0.030, *d* = 1.14; Temperate vs. HW2 2.07 ± 0.63 mL min^−1^ mmHg^−1^, 95% CI: +0.06 to +1.21 mL min^−1^ mmHg^−1^, *P* = 0.034, *d* = 1.04) than in the temperate condition (1.44 ± 0.58 mL min^−1^ mmHg^−1^), due to thermoregulatory‐induced peripheral vasodilation. However, sFA_vc_ only began to decline towards temperate values by day 3 (Temperate vs. HW3 1.75 ± 0.37 mL min^−1^ mmHg^−1^, 95% CI: −0.18 to +0.79 mL min^−1^ mmHg^−1^, *P* = 0.189, *d *= 0.63). No participant showed haemodynamic signs of presyncope or requested premature cessation during the head‐up tilt.

For all days (i.e., temperate, HW1, HW2, HW3), a relationship between the absolute value of ${\bar{T}}_{b}$ with the tilt‐induced change value of sFA_vc_ (*r*_rm_ = −0.60, *P* < 0.001) was found, but no relationships were found with heart rate, MAP, MCA_VCi_ and $P_{\mathrm{ETC}O_{2}}$ (all *P* > 0.05). For only the heatwave days (i.e., HW1, HW2, HW3), no significant relationships were found between the absolute value of ${\bar{T}}_{b}$ and the tilt‐induced change in any cardiorespiratory or cerebrovascular variable (i.e., heart rate, MAP, $P_{\mathrm{ETC}O_{2}}$, sFA_vc_, MCA_VCi_, all *P* > 0.05).

## 4 DISCUSSION

### 4.1 Main findings

The findings of this study in young healthy adult males provide partial support for our primary hypothesis. Three days of simulated heatwave increased resting supine peripheral haemodynamic demand, as indicated by sFA_bf_ and $\mathrm{sFA}D_{O_{2}}$. However, contrary to our expectations, the heatwave did not reduce resting supine regional cerebral blood velocity/flow or $\mathrm{gC}D_{O_{2}}$, nor did it exacerbate orthostatic‐induced reductions in cerebral haemodynamics. Notably, resting supine $\mathrm{gC}D_{O_{2}}$ was increased in the latter 2 days of the heatwave compared to the temperate condition. These divergent cerebral and peripheral haemodynamic responses in males suggest cerebral perfusion and oxygen delivery are prioritised during prolonged heat stress.

### 4.2 Cardiovascular responses at supine rest during a simulated heatwave

The thermoregulatory profile of the 3‐day simulated heatwave, including the moderate heat strain induced on initial exposure and adaptation by the third day of the heatwave, is discussed in detail elsewhere (Ioannou et al., 2021). Our unique protocol enabled the assessment of the cardiorespiratory and cerebrovascular responses to an initial 12 h of continuous heat stress that included both rest and manual working periods (HW1: approx. 9 h rest, 3 h work) for three consecutive days, which contrasts previous investigations that either rapidly induced more severe heat strain (core temperature up to +2.0°C) (Fan et al., 2008; Ganio et al., 2012; Ross et al., 2012; Shibasaki et al., 2020) or accumulated fewer hours (7−14 h) of heat stress across intermittent exposures (Buono et al., 1998; Fujii et al., 2015; Garrett et al., 2012). sFA_bf_ was elevated upon initial exposure and remained elevated throughout the simulated heatwave as hypothesised, demonstrating the responsiveness of the peripheral conduit arteries to increase blood flow to the cutaneous circulation and aid heat dissipation (Amin et al., 2021; Crandall & Wilson, 2015). Despite a rising skin temperature reflecting the extracutaneous demand, blood pressure was maintained by an increased heart rate, although this did not translate to a statistically significant increase in cardiac output. This may be explained by model flow underestimations of stroke volume and cardiac output during heat stress experiments (Shibasaki et al., 2011). Maintenance of blood pressure by increases in cardiac output during subsequent days of the heatwave was likely driven by increased stroke volume as heart rate returned to temperate values by day 3 of the heatwave. As participants had no restrictions on water consumption, a possible explanation for the increase in stroke volume is an expansion of blood volume. Although $P_{\mathrm{ETC}O_{2}}$ was unexpectedly elevated by days 2 and 3 of the heatwave, potentially a result of the confined study setting reducing air ventilation and increasing rebreathing of expired air, previous evidence indicates that hypercapnia does not alter head‐up tilt‐induced reductions in blood flow or head‐up tilt tolerance during heat stress (Lucas et al., 2013; Shibasaki et al., 2020).

Resting supine MCA_v_ did not reduce during the simulated heatwave compared with the temperate condition, contrasting our hypothesis and previous literature (Bain et al., 2013; Fan et al., 2008; Lucas et al., 2018; Nelson et al., 2011; Ogoh et al., 2013; Wilson et al., 2006). Reductions in intracranial cerebral blood velocity during heat stress have largely been attributed to hypocapnia‐induced vasoconstriction caused by hyperventilation, whereas this was not experienced in our cohort and is most likely explained by the less severe heat strain (i.e., smaller increases in *T*_gi_, ${\bar{T}}_{b}$, and ${\bar{T}}_{\mathrm{sk}}$_)_ in our study compared to previous reports (Bain et al., 2013; Fan et al., 2008; Lucas et al., 2018; Wilson et al., 2006). Indeed, in the latter days of the heatwave, our cohort experienced a relative hypercapnia alongside an increased stroke volume, both positively associated with cerebral blood flow during moderate heat stress (Low et al., 2008; Nelson et al., 2011; Ogoh et al., 2014). In the extracranial arteries, comparable ICA_bf_ and VA_bf_ responses to simulated heatwave supports (Ogoh et al., 2013; Shibasaki et al., 2020) and contrasts (Bain et al., 2013; Chapman et al., 2020; Ogoh et al., 2014) previous reports of no regional differences in extracranial blood flow regulation to acute heat stress. When expressed as global cerebral haemodynamics, significant increases in $\mathrm{gC}D_{O_{2}}$ and significant relationships between ${\bar{T}}_{b}$ and $\mathrm{gC}D_{O_{2}}$ were found. Increases in global cerebral haemodynamics during isocapnic passive heat stress have been reported previously and may reflect a matching of a *Q*_10_ coefficient‐related increase in cerebral metabolism (Caldwell et al., 2020). Our data also suggest that $\mathrm{gC}D_{O_{2}}$ was supported by a non‐significant but directionally consistent rise in cardiac output and gCBF and significant increases in Hb. Indeed, the increase in Hb by day 3, in combination with the reduction in heart rate, indicates reduced cardiovascular strain in the latter days of heatwave and may reflect an early expansion of total blood volume, which is typically reported with more prolonged, albeit intermittent, heat exposure (Lundby et al., 2023; Rodrigues et al., 2026). While speculative, these findings raise the possibility that continuous heat exposure may accelerate blood‐volume adaptations from weeks to within days.

### 4.3 Orthostasis during a simulated heatwave

The head‐up tilt stimulus used in this study was reproducible across all temperate and heatwave days, with comparable reductions in stroke volume, cardiac output and $P_{\mathrm{ETC}O_{2}}$ and concomitant increases in heart rate, MAP and TPR. Despite the added cardiovascular strain of an elevated sFA_bf_, cardiac output and ${\bar{T}}_{\mathrm{sk}}$ experienced at rest during the 3‐day simulated heatwave, these maintained hemodynamic responses highlight the resilience of the cardiovascular system under prolonged heat stress. Moreover, head‐up tilt‐induced reductions in MCA_VCi_ were not exacerbated in comparison to temperate conditions despite the additional cardiovascular strain induced by prolonged heat stress. This contrasts with our hypothesis and previous literature that reported greater reductions in cerebral blood velocity with orthostasis during heat stress (Lucas et al., 2018; Wilson et al., 2006). In contrast with the cerebral circulation, the peripheral circulation elevations in resting sFA_VC_ to heat stress were attenuated after head‐up tilt and by day 3 of simulated heatwave sFA_VC_ after the head‐up tilt returned towards temperate values. These divergent cerebral and peripheral responses may highlight an adaptive reciprocal response between the cerebral and peripheral circulations, possibly reflecting an advancement in the peripheral vasoconstrictive reserve capacity to maintain central blood volume and cerebral haemodynamics to preferentially maintain cerebral oxygenation during orthostasis as the simulated heatwave progressed. This reserve capacity of the peripheral vasculature may also suggest that peripheral vascular adaptations with prolonged heat stress are beyond resting adaptations and are of functional importance. For example, the attenuated elevations in sFA_VC_ to a 3‐day simulated heatwave during orthostasis reflect a less effective peripheral heat loss during heat stress whilst standing compared to supine. Reductions in peripheral blood flow during orthostasis to maintain central blood volume and MAP to preferentially preserve cerebral perfusion have been proposed previously (Horiuchi et al., 2021), and may be linked to differences in autonomic control between the cerebral and peripheral vasculature (Koep et al., 2022).

### 4.4 Perspectives and methodological considerations

In line with recent recommendations (Meade et al., 2020), the present study blended physiological measurement with an ecological study design, which is necessary so findings can inform public health guidance. Our finding that cerebrovascular reductions to orthostasis during temperate conditions are not exacerbated by 3 days of consecutive heat stress in young healthy men contrasts with reports using models of acute, rapid and uncompensatable heat stress (Fan et al., 2008; Nelson et al., 2011; Ogoh et al., 2013; Ross et al., 2012; Wilson et al., 2006). Our findings suggest that orthostasis may be well tolerated during more prolonged heat stress akin to heatwaves. Whilst the unchanged cerebrovascular response to head‐up tilt between temperate conditions and prolonged heat stress may indicate that orthostasis is well tolerated, we were unable to determine orthostatic tolerance since the duration of the tilt was limited to 10 min. Previous studies have shown that presyncopal symptoms and/or maximal tolerance occur >15 min (Lind et al., 1968; Minson et al., 1999). This study is part of a wider confinement investigation of the physiological and behavioural thermoregulation responses of young, healthy labour workers (Fisher et al., 2022; Ioannou et al., 2021, 2024). Consequently, it contained a small and homogeneous participant sample, which limits the application of the current findings to young healthy males. Demographic factors such as sex and age influence orthostatic responses during acute heat stress (Meade et al., 2020; Meendering et al., 2005; Schlader et al., 2016), and may also play an important role in the orthostatic responses to prolonged heat stress. Therefore, future research should investigate the physiological responses of females, older adults and heat‐vulnerable individuals to heatwaves, so that climate–health models and public health policy are informed by those at the greatest risk of illness and injury during heatwaves (Meade et al., 2020).

## 5 Conclusions

A 3‐day simulated heatwave increased resting supine leg blood flow and oxygen delivery, and cerebral oxygen delivery in males. The orthostatic‐induced reduction in cerebral haemodynamics was not exacerbated across the heatwave, whereas the heatwave‐induced elevation in supine peripheral (leg) haemodynamics was attenuated during orthostasis, possibly reflecting less effective thermoregulation whilst upright compared to supine. These divergent cerebral and peripheral haemodynamic responses in males suggest cerebral perfusion and oxygen delivery are prioritised during prolonged heat stress.

## AUTHOR CONTRIBUTIONS

Conceptualisation, Urša Ciuha, Leonidas G. Ioannou, Igor B. Mekjavic, and Justin S. Lawley; data curation, Alexander T. Friend, Lydia L. Simpson, and Gabriella M. K. Rossetti; formal analysis, Alexander T. Friend; funding acquisition, Igor B. Mekjavic, and Justin S. Lawley; investigation, Lydia L. Simpson, Carmen Possnig, Jason T. Fisher, Lydia Tsoutsoubi, Konstantinos Mantzios, Urša Ciuha, Leonidas G. Ioannou, Igor B. Mekjavic, and Justin S. Lawley; methodology, Igor B. Mekjavic and Justin S. Lawley; project administration, Igor B. Mekjavic and Justin S. Lawley; resources, Igor B. Mekjavic and Justin S. Lawley; software, Alexander T. Friend; supervision, Samuel J. Oliver, Igor B. Mekjavic, and Justin S. Lawley; validation, Alexander T. Friend; visualisation, Alexander T. Friend and Samuel J. Oliver; writing—original draft preparation, Alexander T. Friend, and Samuel J. Oliver; writing—review and editing, Alexander T. Friend, Samuel J. Oliver, Lydia L. Simpson, Carmen Possnig, Gabriella M. K. Rossetti, Jason T. Fisher, Lydia Tsoutsoubi, Konstantinos Mantzios, Urša Ciuha, Leonidas G. Ioannou, Igor B. Mekjavic, and Justin S. Lawley. All authors have approved the final version of the paper and agree to be accountable for all aspects of the work in ensuring that questions related to the accuracy or integrity of any part of the work are appropriately investigated and resolved. All persons designated as authors qualify for authorship, and all those who qualify for authorship are listed.

## CONFLICT OF INTEREST

The authors declare no conflict of interest. The funder had no role in study design, data collection and analysis, decision to publish, or preparation of the manuscript.

## GENERATIVE AI STATEMENT

No generative AI tools were used in the preparation of this manuscript.

## Data Availability

The data that support the findings of this study are available from the corresponding author upon reasonable request.
